# Three-Dimensional Finite Element Analysis and Characterization of Quasi-Surface Acoustic Wave Resonators

**DOI:** 10.3390/mi12091118

**Published:** 2021-09-17

**Authors:** Wen Chen, Linwei Zhang, Shangshu Yang, Wenhan Jia, Songsong Zhang, Yuandong Gu, Liang Lou, Guoqiang Wu

**Affiliations:** 1 Institute of Technological Sciences, Wuhan University, Wuhan 430072, China; wenwhu@whu.edu.cn (W.C.); ssyang@whu.edu.cn (S.Y.); wh_j2019@whu.edu.cn (W.J.); 2 Shanghai Industrial Technology Research Institute, Shanghai 201899, China; Ethan.Zhang@sitrigroup.com (L.Z.); Songsong.Zhang@sitrigroup.com (S.Z.); Alex.Gu@sitrigroup.com (Y.G.)

**Keywords:** quasi-surface acoustic wave (QSAW) resonator, microelectromechnical systems (MEMS), finite element analysis, aluminum nitride

## Abstract

In this work, three-dimensional finite element analysis (3D FEA) of quasi-surface acoustic wave (QSAW) resonators with high accuracy is reported. The QSAW resonators consist of simple molybdenum (Mo) interdigitated transducers (IDT) on solidly mounted stacked layers of AlN/Mo/Si. Different to the SAW resonators operating in the piezoelectric substrates, the reported resonators are operating in the QSAW mode, since the IDT-excited Rayleigh waves not only propagate in the thin piezoelectric layer of AlN, but also penetrate the Si substrate. Compared with the commonly used two-dimensional (2D) FEA approach, the 3D FEA method reported in this work shows high accuracy, in terms of the resonant frequency, temperature coefficient of frequency (TCF), effective coupling coefficient ($k_{eff}^{2}$) and frequency response. The fabricated QSAW resonator has demonstrated a $k_{eff}^{2}$ of 0.291%, series resonant frequency of 422.50 MHz, and TCF of −23.418 ppm/°C in the temperature range between 30 °C and 150 °C, for the design of wavelength at 10.4 μm. The measurement results agree well with the simulations. Moreover, the QSAW resonators are more mechanically robust than lamb wave devices and can be integrated with silicon-based film bulk acoustic resonator (FBAR) devices to offer multi-frequency function in a single chip.

## 1. Introduction

Surface acoustic wave (SAW) devices are one of the most popular microelectromechnical systems (MEMS) devices at high frequency and super high frequency, which have been widely used in filters, actuators and sensors applications [1,2,3,4]. SAW devices are usually fabricated based on lithium niobate (LiNbO_3_) piezoelectric substrate and have demonstrated excellent performance of high-quality factor (*Q*) as well as large effective coupling coefficient [5,6,7,8]. However, LiNbO_3_ has high electrical impedance at high frequency, resulting in electrical matching challenge with the ultrasound system electronics. LiNbO_3_ suffers from lower phase velocity and poor complementary metal oxide semiconductor (CMOS) compatibility compared with state-of-art aluminum nitride (AlN)-based piezoelectric devices. AlN is a kind of promising piezoelectric material and shows high performance in MEMS piezoelectric devices due to its ultra-high acoustic velocity, high thermal conductivity and good CMOS compatibility [9]. Thus, AlN-based devices offer excellent performance and good manufacturability [10]. AlN is usually deposited as a piezoelectric thin film on a silicon substrate by physical vapor deposition (PVD). Different to the SAW resonator operating in the piezoelectric substrate, acoustic waves excited by interdigitated transducers (IDT) for AlN thin-film-based SAW devices not only propagate in the thin piezoelectric layer of AlN, but also penetrate the Si substrate. Thus, the AlN thin-film-based SAW resonator is more likely to be a kind of quasi-SAW (QSAW) device.

In the early days, numerical calculation is the primary method to investigate SAW resonators. Theoretical studies have been conducted to calculate velocity, coupling coefficient, admittance response and other parameters of SAW transducers roughly [11,12,13]. With the development of computer simulation, finite element analysis (FEA) becomes an important and convenient approach for researchers to optimize designs for SAW devices. Two-dimensional (2D) FEA is a typical method to investigate and design SAW transducers and resonators, which provides a simple and quick way for SAW device simulations [14,15,16,17]. 2D models can be used for quickly obtaining the design parameters, such as frequency, coupling coefficient and phase velocity. However, this method is not accurate, since 2D models miss many key parameters of the devices and the boundary conditions are quite different to the real scenarios. The results by numerical calculation and 2D simulation have difficulties in matching well with the measurement results sometimes. Therefore, both numerical calculation and 2D simulation have limitations in investigating SAW resonators. Simulations based on 3D models can acquire more accurate results, such as series resonance frequency $f_{s}$, parallel resonance frequency $f_{p}$, *Q*, $k_{eff}^{2}$ and other parameters. Simulations based on 3D models are more complicated, but 3D models are closer to real devices than 2D models. Therefore, 3D FEA designs are more accurate and instructive.

In this work, 3D FEA of the solidly mounted stacked layers of AlN/molybdenum (Mo)/Si for QSAW resonators with grounded Mo electrodes are presented. The grounding of the bottom Mo electrode can improve the $k_{eff}^{2}$ of QSAW resonator compared with conventional SAW resonator [18]. 3D FEA simulations are carried out to optimize designs. To verify the accuracy of the 3D FEA approach, key parameters for designing QSAW resonators such as frequency, admittance response, $k_{eff}^{2}$ and temperature coefficient of frequency (TCF) are investigated and discussed in both simulation and experiment.

## 2. Device Design

**Figure 1:**
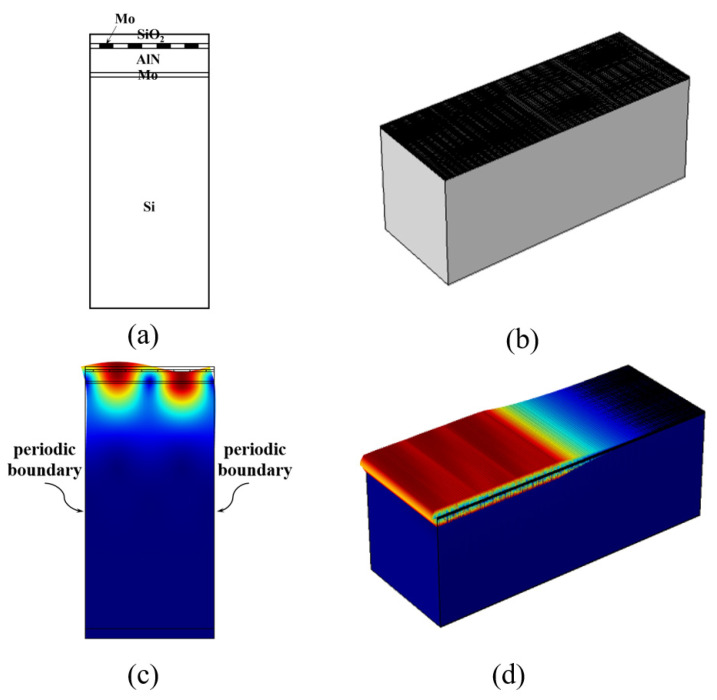
Illustration of 2D and 3D FEA models and simulated displacement profiles of the QSAW resonators: (**a**) Simplified 2D FEA model structure. (**b**) 3D FEA model structure. (**c**) 2D FEA displacement profile. (**d**) 3D FEA displacement profile.

The QSAW resonators consist of Mo IDTs and reflectors laid on the solidly mounted stacked structure of AlN/Mo/Si layers, as shown in Figure 1. Both 2D and 3D FEA simulations are conducted in COMSOL Multiphysics (COMSOL Multiphysics 5.4, COMSOL AB, Stockholm, Sweden), in order to compare their differences. The QSAW resonators are designed with four different wavelengths, λ, of 10.4 μm, 10 μm, 9.6 μm and 9.2 μm. In this work, split IDT design, rather than typical IDT structure, is adopted to reduce the insertion loss and improve the frequency stability [19]. The split IDT is composed of a grating structure with interval of λ/8 and a center-to-center distance of λ/4. The aperture length is designed as 50λ. The IDT fingers number and reflector gratings number are 60 pairs and 80 pairs, respectively. The simulated displacement profiles of the 2D and 3D models are shown in Figure 1. In 2D simulations, periodic boundary conditions are applied on the left and right sides of the model, as shown in Figure 1c. For 3D simulations as shown in Figure 1b, the model is anti-symmetric in the center plane (the leftmost plane in Figure 1b). The displacement profile in the 3D FEA model is presented in Figure 1d.

**Figure 2:**
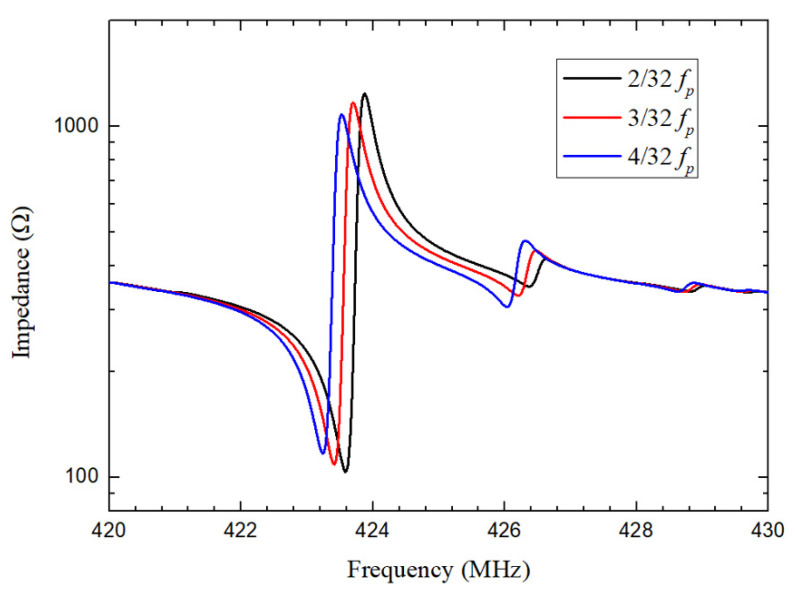
Simulated impedance curves of QSAW resonator with λ of 10.4 μm for different spaces of 2λ/32, 3λ/32 and 4λ/32.

In the simulations, space between IDT fingers area and reflector gratings area is varied to optimize designs of the QSAW resonators. The impedance responses of QSAW resonators with λ of 10.4 μm for different spaces of 2λ/32, 3λ/32 and 4λ/32 are obtained in 3D FEA simulations. As shown in Figure 2, the impedance response of resonator with space of 2λ/32 is better than that of other two cases. Moreover, spurious mode amplitude at about 426 MHz is obviously smaller for the design with space of 2λ/32. In other words, the signal response at series resonance frequency is heightened and undesired spurious mode is suppressed for optimized space. Smaller space between IDT fingers and reflector gratings will reflect more surface acoustic waves back to IDT fingers area, resulting in stronger frequency response at series resonance frequency and less spurious modes. However, the patterning of tiny space around hundreds of nanometers is extremely challenging in lithographic process due to the critical dimension limitations of the tool used. Therefore, the space is set as 2λ/32 in this work. Table 1 lists the detailed parameters of the QSAW resonators. In simulations, the material parameters of all layers included in models are presented in Table 2, which are adopted from [20,21].

## 3. Device Fabrication

**Figure 3:**
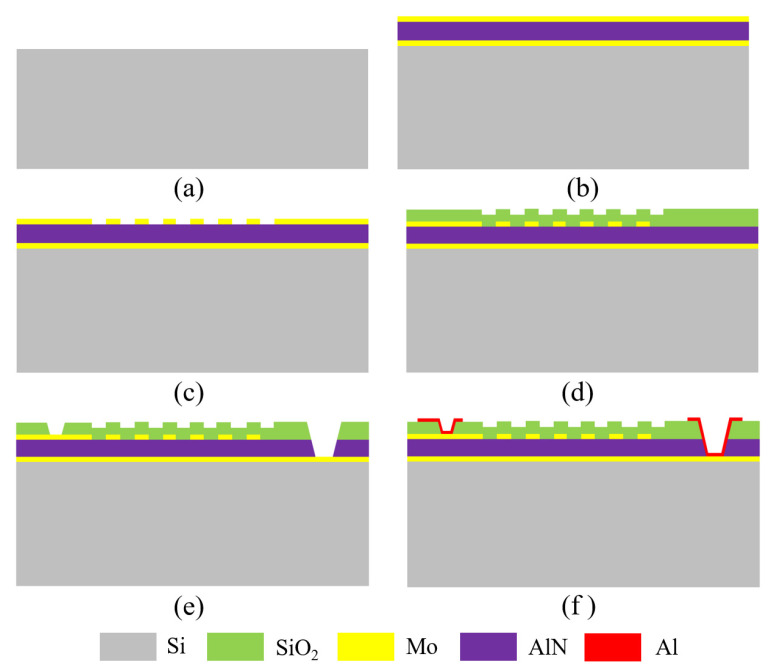
Process flow of the QSAW resonators. (**a**) Starting with a Si wafer, (**b**) Mo/AlN/Mo layers deposition, (**c**) Top Mo layer patterning to define IDT fingers and reflector gratings, (**d**) Top oxide deposition, (**e**) Contact vias opening to access the top and bottom electrodes, (**f**) Al deposition and patterning to form the bonding pads and connection lines.

Figure 3 illustrates the fabrication process of the QSAW resonators. The fabrication starts with a 500-μm-thick Si wafer as substrate. Next, a 0.2-μm-thick Mo thin film is deposited on the wafer as bottom electrode, and followed by 1-μm-thick c-axis oriented AlN piezoelectric thin film and another 0.2-μm-thick Mo thin film depositions using sputtering. Then, the top Mo layer is patterned to define IDT fingers and reflector gratings. After that, a 0.5-μm-thick oxide is deposited using plasma enhanced chemical vapor deposition (PECVD) on the top Mo layer to prevent the top electrode from being oxidized. Electric contact vias are defined and patterned to access the top and bottom Mo electrodes, respectively. Finally, a 1-μm-thick aluminum layer is deposited and patterned to form the bonding pads and connection lines.

**Figure 4:**
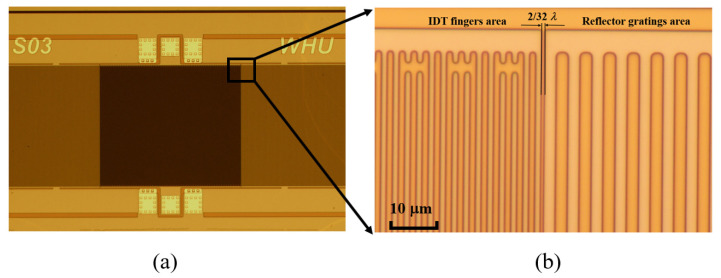
Optical microscope images of a fabricated QSAW resonator: (**a**) overall view of resonator, (**b**) zoom-in view of space between IDT fingers and reflector gratings.

The optical microscope images of a fabricated QSAW resonator are shown in Figure 4. The IDT fingers are located at the center area, and the reflector gratings are located at two flanks. The IDT fingers and reflector gratings are clearly visible as displayed in Figure 4b. The space between IDT fingers and reflector gratings is defined as 2λ/32.

## 4. Results

**Figure 5:**
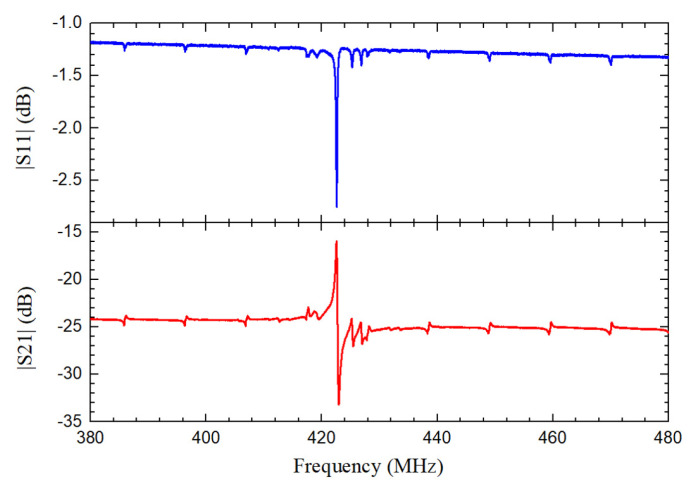
Measured S11 and S21 curves of the QSAW resonator with λ of 10.4 μm in a wide frequency range from 380 MHz to 480 MHz.

Scattering (*S*) parameters of the fabricated QSAW resonators are characterized by a Keysight vector network analyzer in air with an open chamber probe station at room temperature. The devices are measured with ground-signal-ground (GSG) probes after conducting a standard short-open-load-through calibration using a standard calibration substrate. The measured S11 and S21 curves for fabricated resonator with λ of 10.4 μm in a wide frequency range from 380 MHz to 480 MHz are shown in Figure 5. As displayed in Figure 5, the series resonance frequency $f_{s}$ and the parallel resonance frequency $f_{p}$ are 422.50 MHz and 422.99 MHz, respectively. The insertion loss is −16.54 dB and *Q* is 1616 at its series resonance frequency.

**Figure 6:**
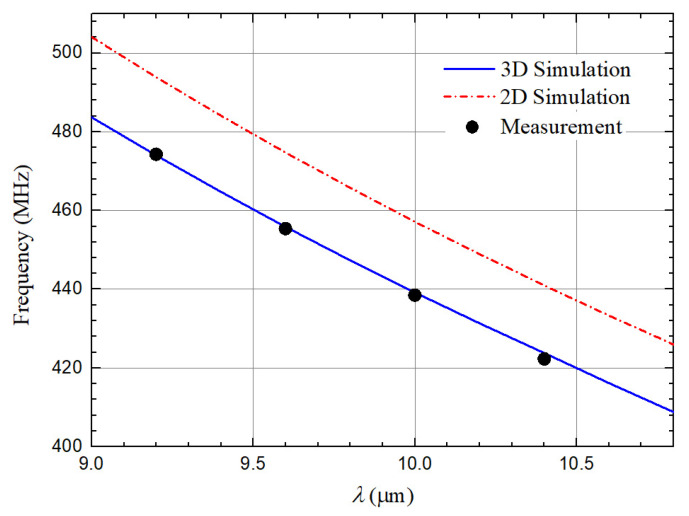
2D, 3D FEA simulated and measured frequencies with different λ.

**Figure 7:**
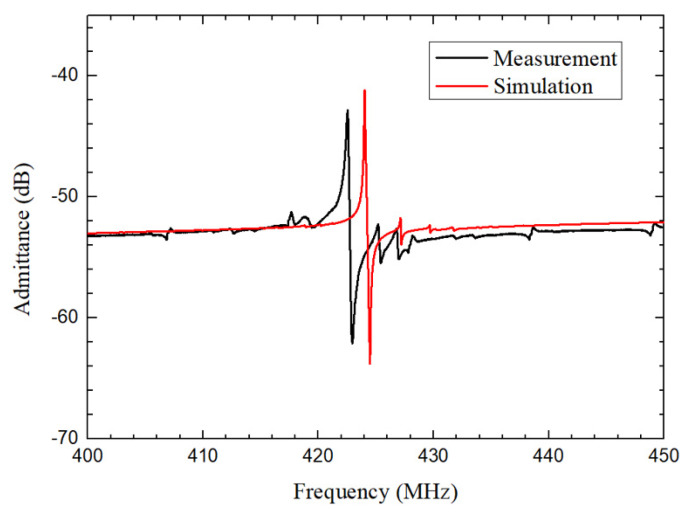
Measured and simulated admittance responses of the QSAW resonator with λ of 10.4 μm in a frequency range from 400 MHz to 450 MHz.

λ is set as 10.4 μm, 10 μm, 9.6 μm and 9.2 μm to verify the frequency accuracy of 3D FEA simulation. Comparison of the simulated and measured $f_{s}$ under different λ is illustrated in Figure 6. It should be noted that the frequencies of fabricated resonators are very consistent with the 3D FEA simulated results. Although there is significant deviation between measured results and 2D FEA simulated results. This indicates that the 3D FEA simulations show higher accuracy in obtaining the simulated frequencies compared to the 2D FEA approach. The dimensions and configuration of 3D model are consistent with the practical scenario of the fabricated QSAW resonator. However, a simplified mode is used in 2D FEA approach and hence the setting of the boundary conditions is not consistent with the practical situation, resulting in large deviations between the simulation and measurement. Measured admittance response of the QSAW resonator with λ of 10.4 μm is also analyzed and compared with simulation result. Measured and simulated admittance responses of the above mentioned QSAW resonator in a frequency range from 400 MHz to 450 MHz are presented in Figure 7. The simulated frequency response is almost coincided with measured frequency response. However, simulated admittance response at series resonance frequency is slightly better than measurement result due to higher *Q* used in the simulation. This is because some energy loss mechanisms such as the phonon-phonon interaction loss are not considered in simulations. There is a slight difference of series and parallel resonance frequencies between the simulated values and the measurement results, as displayed in Figure 7. The possible reason is that the material parameters in 3D FEA model are different to the real parameters of the materials used.

$k_{eff}^{2}$ of QSAW resonator is also investigated and compared in both simulation and experiment. $k_{eff}^{2}$ is defined as following [22]:

(1) $$\begin{array}{c} k_{eff}^{2}=\frac{\pi^{2}}{4}\frac{f_{s}}{f_{p}}\frac{f_{p}-f_{s}}{f_{p}}, \end{array}$$

**Figure 8:**
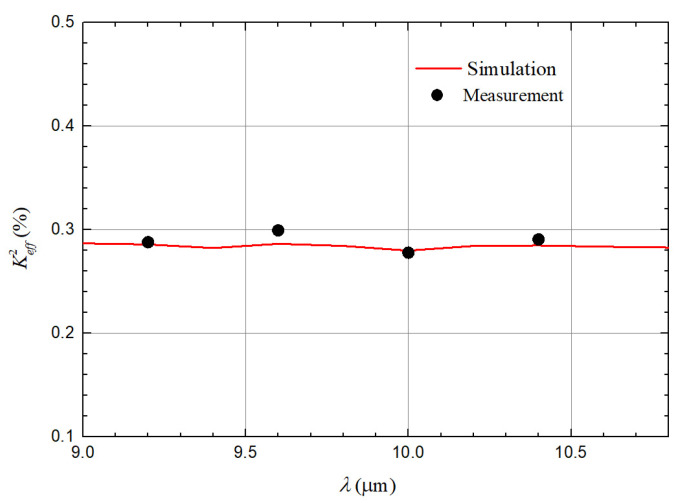
Measured and simulated $k_{eff}^{2}$ of the QSAW resonator with different λ.

The simulated and measured $k_{eff}^{2}$ of QSAW resonators with different λ are displayed in Figure 8. The measured and simulated results are extremely close, which indicates the high accuracy of 3D simulation.

**Figure 9:**
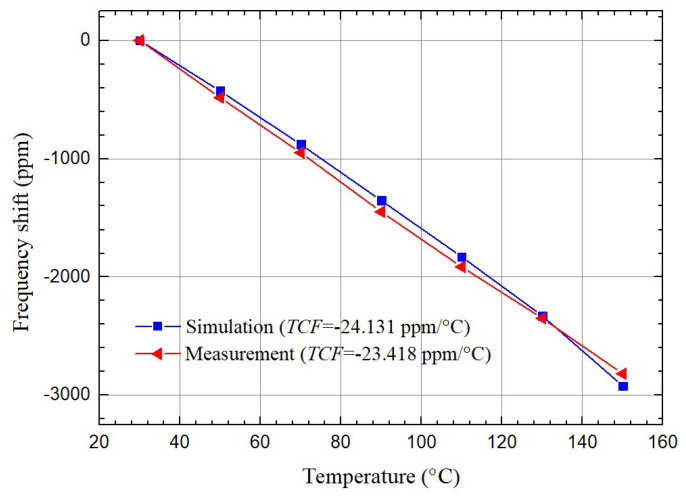
Measured and simulated *TCF*s of the QSAW resonator with λ of 10.4 μm in a temperature range from 30 °C to 150 °C.

The *TCF*s of QSAW resonators with λ of 10.4 μm, 10 μm, 9.6 μm and 9.2 μm are measured in a temperature range from 30 °C to 150 °C. The *TCF* measurements are conducted in a cryogenic probe station. The simulated and measured *TCF*s of QSAW resonator with λ of 10.4 μm are presented in Figure 9. The simulated and measured *TCF*s are −24.131 ppm/°C and −23.418 ppm/°C, respectively. It is noted there is a small difference between simulated and measured results, due to the imperfection of the material parameters and the process tolerances in the fabrication.

The measured and 3D FEA simulated results with relative errors are presented in Table 3. The simulated frequencies for QSAW resonators are very close to measured results. The measured and 3D FEA simulated results of $k_{eff}^{2}$ and *TCF* are close for QSAW resonators with λ of 10.4 μm, 10 μm and 9.6 μm. However, there is a little deviation between simulated and measured results of *TCF* for QSAW resonator with λ of 9.2 μm. As illustrated in the table, the negligible differences between simulation and measurement results indicate high accuracy of the 3D FEA simulations for the QSAW resonator.

## 5. Conclusions

In this work, a 3D FEA approach for designing QSAW resonators with high accuracy is presented. To validate the accuracy of the 3D FEA method, the QSAW resonators with λ of 10.4 μm, 10 μm, 9.6 μm and 9.2 μm are designed, fabricated and characterized. The reported QSAW resonator is composed of a solidly mounted stacked structure of AlN/Mo/Si layers with split Mo IDT fingers laid on the surface. The measured frequencies of QSAW resonators for different λ are consistent with 3D simulation results and have significant deviations with 2D simulation results, indicating high accuracy of 3D FEA simulation. The admittance response, $k_{eff}^{2}$ and *TCF* are also investigated and compared in both simulations and measurements. The measurement results are in good agreement with 3D FEA simulation values, which indicates high accuracy of 3D FEA simulation. Key parameters of QSAW or SAW resonators can be obtained accurately through simulations, which is crucial for designing QSAW or SAW resonators with high performance in practical applications.

## Figures and Tables

**Table 1:** Design parameters of QSAW resonators.

IDT fingers number	60 pairs
Reflector gratings number	80 pairs
Wavelength (λ)	10.4 μm
	10 μm
	9.6 μm
	9.2 μm
Space	2λ/32
Aperture length	50λ

**Table 2:** Material parameters of all layers used in the simulations.

		AlN	Si	SiO_2_	Mo
Elastic constants, $c_{ij}$ (GPa)	$c_{11}$	410.06	165.6	70	329
	$c_{12}$	100.69	63.9		
	$c_{13}$	83.82			
	$c_{33}$	386.24			
	$c_{44}$	100.58	79.5		
	$c_{66}$	154.70			
Relative permittivity, $\varepsilon_{ij}$	$\varepsilon_{31}$	9		4.2	
	$\varepsilon_{33}$	11		4.2	
Density, ρ (kg/m^3^)		3260	2329	2200	10,200
Piezoelectric stress constants,	$e_{15}$	−0.48			
$e_{ij}$ (C/m^2^)	$e_{31}$	−0.58			
	$e_{33}$	1.55			

**Table 3:** Comparison of simulation and measurement results.

λ (μm)		10.4	10	9.6	9.2
Frequency	Simulation	423.18	439.17	455.84	473.99
(MHz)	Measurement	422.50	438.68	455.39	474.27
Error		0.16%	0.112%	0.098%	0.059%
$K_{eff}^{2}$	Simulation	0.285%	0.280%	0.286%	0.286%
	Measurement	0.291%	0.278%	0.299%	0.288%
Error		1.99%	0.732%	4.42%	0.743%
TCF	Simulation	−24.131	−24.616	−25.088	−25.094
(ppm/°C)	Measurement	−23.418	−25.102	−26.117	−28.056
Error		2.69%	1.97%	4.10%	11.804%

## Abbreviations

*Q*	Qulity factor,
$k_{eff}^{2}$	Effective coupling coefficient,
λ	Wavelength,
*c*	Elastic constant,
ε	Relative permittivity,
ρ	Density,
*e*	Piezoelectric stress constant,
$f_{s}$	Series resonance frequency,
$f_{p}$	Parallel resonance frequency
